# Supporting the Primary Outcomes of the Mirai Trial for the Adjunctive Digital Therapeutic Rejoyn (CT-152) in the Treatment of Major Depressive Disorder: Meaningful Change Analysis in the Montgomery-Åsberg Depression Rating Scale

**DOI:** 10.2196/85037

**Published:** 2026-09-21

**Authors:** Stacie Hudgens, Intan Purnajo, Lysbeth Floden, Steve Hwang, Jessica Ash, Brian Rothman, Austin Speier, Ainslie Forbes

**Affiliations:** 1 Clinical Outcomes Solutions Ltd., Tucson, AZ, United States; 2 Otsuka Pharmaceutical Development & Commercialization, Inc., 508 Carnegie Center Drive, Princeton, NJ, 08540, United States, 1 609 524 6788; 3 Click Therapeutics, Inc., New York, NY, United States

**Keywords:** cognitive therapy, digital health intervention, meaningful within-patient change, patient-reported outcomes, psychopharmacological augmentation therapy

## Abstract

**Background:**

Rejoyn (CT-152) is a prescription digital therapeutic (DTx) adjunct to antidepressive medication authorized for patients with major depressive disorder. To better understand the patient benefit of DTx and other treatment modalities, current regulatory standards support the use of modern psychometric methods to interpret the clinical meaningfulness of treatment effects for patients. In the primary analysis of Rejoyn from the pivotal phase 3 Mirai trial (NCT04770285), Rejoyn showed a broad risk-to-benefit profile as demonstrated on multiple clinician- and patient-rated scales, including the primary efficacy outcome measure, the Montgomery-Åsberg Depression Rating Scale (MADRS). These findings supported US Food and Drug Administration authorization of Rejoyn as a prescription DTx. However, the clinical relevance of these changes in the MADRS score is not immediately interpretable in clinical practice. Here, we present the results from several post hoc analyses of the Mirai trial data to support the interpretation of clinically meaningful treatment differences on clinician- and patient-reported change in depressive symptoms.

**Objective:**

This study had two main objectives: (1) establish threshold parameters that allow for clinically meaningful interpretation of the Mirai results in clinical practice, based on the clinical trial end points of change from baseline in depressive symptoms, and (2) apply this threshold in a responder and sensitivity analysis in the intent-to-treat (ITT) population (which is more frequently reported in pharmacological trials) to further support the interpretation of change in unblinded analysis of the Mirai results.

**Methods:**

For the Mirai meaningful within-patient change (MWPC) analysis, anchor-based methods were used to define an MWPC threshold by exploring the associations between the MADRS and the clinician-rated Clinical Global Impression-Severity Scale (CGI-S) and the patient-reported Patient Health Questionnaire 9-Item Scale (PHQ-9). Additional post hoc efficacy analyses (including that of responders) are reported for the ITT population.

**Results:**

Using the MWPC thresholds of 8 and 10 points (derived with the CGI-S and PHQ-9 as anchor measures, respectively), the distribution of MADRS responders favored the Rejoyn group over the sham group, and the Rejoyn group had 24%‐47% higher odds of meaningful improvement on the MADRS. Post hoc ITT analyses also favored the Rejoyn group over the sham group for response rates and PHQ-9 and CGI-S score change from baseline.

**Conclusions:**

Results are consistent with the primary findings of the Mirai trial, supporting the efficacy of Rejoyn as an adjunctive treatment to antidepressive medication monotherapy for adults with major depressive disorder. The MWPC analyses offered a measure of meaningful change on the MADRS, providing a framework of clinical meaningfulness for the Mirai trial findings.

## Introduction

Fewer than half of patients with major depressive disorder (MDD) achieve remission after first-line antidepressive medication [1-3]. Additionally, patients experience barriers to continuous MDD treatment, including inability to access mental health care due to a shortage and uneven distribution of mental health care providers, costs of care, and stigma associated with receiving care for mental disorders [4-6].

Rejoyn (CT-152) is a prescription digital therapeutic (DTx) that received US Food and Drug Administration (FDA) Center for Devices and Radiological Health clearance on March 30, 2024 [7]. It is intended for the treatment of MDD symptoms as an adjunct to clinician-managed outpatient care for adult patients with MDD aged 22 years or older who are receiving antidepressive medication monotherapy [8]. Rejoyn includes 3 components: (1) cognitive emotional training (Emotional Faces Memory Task [EFMT]), (2) cognitive behavioral therapy (CBT)–based lessons, and (3) personalized text messages [8,9].

The FDA clearance of Rejoyn was supported by the results of the pivotal phase 3 Mirai clinical trial (participants with MDD randomly assigned to Rejoyn or sham app adjunctive treatment; NCT04770285). Efficacy outcomes included change from baseline to the end of the 6-week treatment period in (1) the primary outcome Montgomery-Åsberg Depression Rating Scale (MADRS) and (2) the secondary outcomes Clinical Global Impression-Severity Scale (CGI-S), Patient Health Questionnaire 9-Item Scale (PHQ-9), and Generalized Anxiety Disorder 7-Item Scale (GAD-7) [9]. Prespecified outcomes for the modified intent-to-treat (mITT) sample and supportive analysis of the primary outcome in the intent-to-treat (ITT) sample in Mirai were previously reported [9]. Briefly, Rejoyn resulted in improvements in depressive symptoms compared with sham on all scales, regardless of scale type or rater, and safety data revealed a favorable risk-benefit profile [9].

The MADRS, used for the primary outcome in Mirai, is included in the FDA Center for Drug Evaluation and Research Clinical Outcome Assessments Compendium, which is a reference document that is intended to be a starting point for drug developers when considering a clinical outcome assessment for use in clinical trials [10]. Per the FDA’s Patient-Focused Drug Development Draft Guidance documents 3 and 4, it is necessary to apply modern psychometric methods to interpret score change in day-to-day practice [11-13]. In the Patient-Focused Drug Development Draft Guidance 4, the FDA recommends the use of multiple anchors when calculating meaningful within-patient change (MWPC) [13]. Anchors should be selected from scales that are established, valid measures of the outcome in question, and that are easier to interpret than the target scale (in the case of the Mirai study, the MADRS was the target scale) [14].

Because Rejoyn represents a new treatment modality, post hoc efficacy and responder analyses of the Mirai results were conducted to support the interpretation of treatment differences on clinician- and patient-reported change in depressive symptoms. This study had two main objectives: (1) to establish thresholds for the MADRS that allow for clinically meaningful interpretation of the Mirai results in day-to-day practice and (2) to apply these thresholds in a responder and sensitivity analysis in the ITT population (which is more frequently reported in pharmacological trials) to further support the interpretation of change in unblinded analysis of the Mirai results [8,13,15,16].

For the Mirai MWPC analysis, an anchor-based approach was applied to define meaningful score change, in alignment with current regulatory standards [11-13]. Associations were explored between the primary end point MADRS and the clinician-rated CGI-S and the patient-reported PHQ-9, which were used for anchoring; the CGI-S and PHQ-9 were chosen, as meaningful treatment differences are more easily interpretable or already known for these scales [8,15,16]. The derived MWPC threshold was then used to evaluate how patients responded to Rejoyn versus sham in both the mITT and ITT populations. While most of the primary Mirai outcomes were analyzed in the mITT to stringently establish the effectiveness of Rejoyn, outcomes in this population may not be easily translated to the broader patient population seen in daily clinical practice [8,9,13,15,16]. To ease interpretation in daily practice and provide a closer frame of reference to traditional pharmacological clinical trial results, further post hoc efficacy analyses of the ITT population are also presented [8,15,16]. Together, these analyses were undertaken to provide a better understanding of how the results of the pivotal Mirai trial compare with other reported study results on treatments for MDD [8,15,16].

## Methods

### Study Design

The full trial methods [17], including the full schedule of assessments [17] and primary analysis results [9], were previously described. A CONSORT (Consolidated Standards of Reporting Trials) checklist is included as Checklist 1. Briefly, Mirai (ClinicalTrials.gov NCT04770285) was a phase 3, multicenter, randomized, blinded, sham-controlled trial conducted remotely [17]. The trial lasted for 13 weeks, including a 3-week screening period, a 6-week treatment period, and a 4-week extension period [17]. The primary efficacy outcome was change in MADRS score from baseline to week 6 [17]. Other efficacy outcomes included change in score from baseline to week 6 for the CGI-S and PHQ-9 and week 6 MADRS partial response rate (≥30% but <50% reduction from baseline), full response rate (≥50% reduction from baseline), and remission (≥50% reduction from baseline and score ≤10) [17].

### Participants and Recruitment

Data were collected from 368 participants across 37 trial sites in the United States. All participants were recruited on an outpatient basis. Key inclusion criteria included participants aged 22‐64 years; a primary diagnosis of MDD based on the *Diagnostic and Statistical Manual of Mental Disorders* (DSM), Fifth Edition; a score of 18 or higher on the 17-item Hamilton Rating Scale for Depression; inadequate response to an adequate antidepressive medication trial in the current episode (inadequate response was defined as <50% reduction in depression symptom severity, and an adequate trial was defined as ≥6 weeks at a minimum therapeutic dose [or higher], both evaluated per the Massachusetts General Hospital-Antidepressant Treatment Response Questionnaire); and receiving a stable dose of antidepressive medication monotherapy for more than 4 weeks that could be maintained during the study [17]. Key exclusion criteria included an inadequate response to more than 1 adequate trial of antidepressive medication for the current major depressive episode, prior treatment with psychopharmacological augmentation therapy for depression, psychotherapy within 90 days before screening, and prior failure of an adequate course of CBT [17]. Eligible participants continued their current antidepressive medication and were randomly assigned 1:1 to Rejoyn or a sham app [17].

### Study Intervention

Rejoyn is a smartphone app that delivers a treatment with three components: (1) EFMT exercises (a modified N-back working memory task asking the participant to recall the emotional expression from a series of faces); (2) brief CBT-based lessons, each paired with an out-of-app activity or guided audio psychotherapy exercise targeting the most common MDD symptoms; and (3) text messaging to reinforce the lessons and encourage engagement [17]. The sham app used the shapes memory task, an emotionally neutral working memory task matched for time and attention to EFMT, along with text messaging [17]. Each Rejoyn session during the 6-week treatment period (18 total, 3 per week) consisted of an EFMT exercise and a brief CBT-based lesson with an activity; the 18 sham app sessions consisted of only a Shapes Memory Task exercise [17]. In Mirai, participants were scheduled to complete 3 sessions per week for 6 weeks [17].

### Ethical Considerations

The Mirai trial was conducted in accordance with local laws, the International Conference on Harmonization Good Clinical Practice guidelines, and the Declaration of Helsinki. The protocol was reviewed and approved by the governing institutional review board (IRB) for each investigational site [17]. Of the 54 study sites, IRB services were provided by Advarra (approval 00000971) for 53 sites, and WCG IRB (approval 1-1377994-1) for 1 site [17]. IRB approvals were granted between November 2020 and January 2021 [17]. All participants were asked to provide consent to participate in this study before enrollment and agree to its privacy policy and terms of service required for the remote collection of their information [17]. All information collected in the trial was considered confidential and was only used in accordance with the protocol [17]. Participants were identified only by a unique identification number in eSource [17]. However, regulatory officials or sponsor personnel could be allowed access to records, consistent with local requirements [17]. Participants could withdraw from the trial at any time without justification [17]. Participants received a stipend to help cover the costs associated with being in the study [17]. Participants also had the option to have their antidepressant medication provided to them by the sponsor at no charge for the length of the study [17].

### Assessment Instruments

#### Montgomery-Åsberg Depression Rating Scale

The MADRS is a clinician-reported outcome designed to measure the severity of depression and comprises a series of questions aimed at evaluating various symptoms of depression [18]. The scale is composed of 10 items, each of which is based on the patient’s reported experiences over the past week and scored from 0=symptom not present or normal to 6=severe or continuous presence of the symptom, for a total possible score of 60 [18]. Higher scores indicate more severe depressive symptoms [18]. In Mirai, the MADRS was completed by an independent blinded central rater and was assessed at weeks 2, 4, and 6 [17].

#### Clinical Global Impression-Severity Scale

The CGI-S is designed to capture the clinician’s impression of the patient’s current clinical status without delving into specific symptoms or diagnostic criteria [19]. It provides a global snapshot of how severe the clinician perceives the patient’s condition to be at the time of assessment [19]. The scale ranges from 1 to 7, with 7 being the “most extremely ill” or highest severity of MDD [19]. In Mirai, the CGI-S was completed by the site clinician and was assessed at weeks 2, 4, and 6 [17].

#### Patient Health Questionnaire 9-Item Scale

The PHQ-9 is a self-reported questionnaire designed to assess the severity of symptoms of MDD [20]. The PHQ-9 consists of 9 questions based on the diagnostic criteria for MDD in the DSM, Fourth Edition [20]. Each question corresponds to 1 of the 9 criteria for MDD and is scored on a scale from 0=not at all to 3=nearly every day [20]. The scores for each item are summed up to yield a total score ranging from 0 to 27 [20]. Higher scores indicate a greater severity of MDD symptoms [20]. In Mirai, the PHQ-9 was completed by the participant and was assessed at weeks 4 and 6 [17].

### Statistical Analysis

#### Overview

Post hoc MWPC calculations and responder analyses were conducted in the mITT sample (participants receiving ≥1 treatment session with both baseline and ≥1 postbaseline MADRS assessments).

A sensitivity analysis for the responder analysis and the post hoc efficacy analyses were conducted in the ITT sample (all participants who were randomly assigned). The standardized effect size (SES) was used to judge the magnitude of difference for MWPC and responder analyses. The SES was calculated as the mean score change divided by the SD of the baseline scores and judged based on Cohen recommendations: small change (SES=0.20), moderate change (SES=0.50), and large change (SES=0.80) [21]. *P* values were summarized, although they were descriptive in nature and unadjusted for multiplicity. Post hoc efficacy end points were tested at a nominal 0.05 level (2-sided) without adjusting for multiplicity [9].

Analyses were performed with SAS (version 9.4; SAS Institute, Inc).

#### MWPC Threshold Calculation

Anchor-based methods were applied using blinded, treatment-agnostic data to identify the parameters and thresholds for defining MWPC on the MADRS. The anchor-based analyses used 2 anchors, the CGI-S (primary anchor) and the PHQ-9. Anchors are typically considered viable if the correlation coefficient is 0.40 or above, with the measure of interest (MADRS for this analysis) to be used in the anchor-based analysis.

Polyserial correlation coefficients for baseline scores and change from baseline scores were assessed to inform the suitability of the CGI-S. Spearman correlation coefficients were assessed to inform the suitability of the PHQ-9 and were used between the PHQ-9 and the MADRS because both scores were assumed to be continuous.

To determine the MWPC threshold, change within each uncollapsed (ie, unchanged) anchor group on the CGI-S was assessed from baseline to week 6. These groups were 1-point worsening (ie, increase in baseline score by 1 point), no change, 1-point improvement (ie, decrease in baseline score by 1 point), 2-point improvement, 3-point improvement, and 4-point improvement. The CGI-S is a single-item (ordinal) global scale, in which each CGI-S total score also corresponds to a category of disease severity as follows: 1 point is “normal,” 2 points is “borderline,” 3 points is “mild,” 4 points is “moderate,” and 5 points is “marked” [19].

PHQ-9 scores are assumed to be continuous, requiring categorization. Additionally, collapsed (ie, consolidated) anchor groups were used to assess PHQ-9 change from baseline to week 6 to derive the MWPC threshold. Because collapsing is typically conducted when the group sample sizes are not adequate (n<5), the threshold derived from the PHQ-9 anchor was not used for primary interpretation. Scores were collapsed using a previously established 6-point change using the following categories: improvement (≥6 points), no change, and worsening (≥6 points) [15]. Mean change, SD, 95% CI, median change, SES, and *P* value statistics were calculated for each change score within each anchor group.

The chosen MWPC thresholds were large enough to exclude participants who had no change, but small enough to include participants who had meaningful change. Significance associated with within-patient change was evaluated using paired 2-tailed *t* tests on the MADRS for MWPC threshold derivation with each anchor category.

Anchor-based meaningful change for the MADRS was also evaluated graphically with cumulative distribution function (CDF) and probability density function (PDF) curves. The CDF curves allowed assessment of the degree to which the anchor categories were separated by the index measure score, while the PDF curves allowed a graphical representation of the central tendency and variability of the scores within each anchor category.

Between-group change comparing adjacent change categories was also evaluated to estimate group-based minimal important differences. Comparisons were evaluated for all adjacent anchor groups using a 2-tailed group *t* test. The average difference in score at week 6 between adjacent groups on both the CGI-S and PHQ-9 was calculated and presented with SE, 95% CI, SES, and *P* value.

#### Responder Analyses

Once determined, the MWPC thresholds for the MADRS were applied to unblinded, comparative responder analyses. Proportions of responders were compared by treatment arm as raw proportions evaluated by a chi-square test or in a logistic regression framework.

An exploratory responder analysis to assess the convergence of efficacy data was also conducted on the PHQ-9 using the previously established 6-point threshold [15]. Patients were classified as responders according to the following definitions: improved—the change in score from baseline to week 6 improved in magnitude by an amount greater than or equal to the magnitude of the MWPC threshold; and not improved—the change in score from baseline to week 6 did not improve in magnitude by an amount greater than or equal to the magnitude of the MWPC threshold. As a sensitivity analysis, responder analyses were also conducted on the ITT population. Although proportions of responders could not be generated for the multiply imputed dataset, summary statistics were provided. CDF curves describing the proportion of patients at each level of change were generated using MADRS change from baseline to week 6, by treatment arm, to support interpretation and specificity to the given thresholds.

#### Exploratory Analysis of MADRS Difference Between Treatment Arms

In an exploratory analysis, the mean change and SES of change were estimated using raw MADRS scores to provide a full picture of the clinically meaningful benefit of Rejoyn.

#### Efficacy Analyses in the ITT Population

For each outcome, the efficacy analysis was performed for the ITT sample using an analysis of covariance model. The model included fixed variables for treatment group, treatment-by-visit interaction, and pooled trial center to assess data through multiple imputation for change from baseline to week 6 in the MADRS total score, with baseline MADRS-by-visit as a covariate. Responder analysis using the per-protocol definition for response (full response: ≥50% reduction in MADRS score from baseline and partial response: reduction of ≥30% but <50%) was performed using Cochran-Mantel-Haenszel testing, controlling for site effect to obtain estimated relative risk with 95% CI and *P* values.

## Results

### Participants

Participant disposition is listed in Table 1. The ITT sample included 194 participants in the Rejoyn group and 192 in the sham group. The mITT analysis set included 177 participants in each group. Full demographic characteristics for the ITT and mITT samples have been published [9]. In the ITT sample, the mean age was 42.6 (SD 12.1) years, and most participants were female (332/386, 86%) and White (301/386, 78%); mean scale baseline scores were: MADRS: 28.4 (SD 6.0), PHQ-9: 15.3 (SD 4.7), and CGI-S: 4.3 (SD 0.5) [9]. In the mITT sample, the mean age was 42.4 (SD 12.1) years, and most participants were female (306/354, 86.4%) and White (276/354, 78%); mean scale baseline scores were: MADRS: 28.5 (SD 6.0), PHQ-9: 15.2 (SD 4.7), and CGI-S: 4.3 (SD 0.5) [9].

**Table 1.** Participant disposition in the Mirai study.

Disposition	Rejoyn (n=194), n (%)	Sham app (n=192), n (%)	Total (N=386), n (%)
Randomly assigned to treatment^a^	194 (100)	192 (100)	386 (100)
Safety analysis sample^b^	187 (96.4)	186 (96.9)	373 (96.6)
Efficacy analysis sample^c^	177 (91.2)	177 (92.2)	354 (91.7)
Completed treatment	165 (85.1)	164 (92.2)	329 (85.2)
Discontinued	29 (14.9)	28 (14.6)	57 (14.8)
Lost to follow-up	11 (5.7)	8 (4.2)	19 (4.9)
Withdrew	9 (4.6)	8 (4.2)	17 (4.4)
Protocol deviation	6 (3.1)	6 (3.1)	12 (3.1)
Noncompliance	3 (1.5)	3 (1.6)	6 (1.6)
Lack of efficacy	0 (0)	1 (1)	1 (0.3)
Technical problem	0 (0)	1 (1)	1 (0.3)
Other	0 (0)	1 (1)	1 (0.3)

a Intent-to-treat sample (all participants who were randomly assigned to treatment).

b Safety analysis sample (participants who received ≥1 treatment session).

c Modified intent-to-treat sample (participants who received ≥1 treatment session with a Montgomery-Åsberg Depression Rating Scale [MADRS] assessment at baseline and ≥1 MADRS assessment after baseline).

### MWPC Threshold Calculation

Using the CGI-S as an anchor, mean changes in MADRS scores from baseline to week 6 monotonically decreased for all categories from worsening to adjacent improvement groups (Table 2). The correlation between change on the MADRS and change on the CGI-S (change correlation) at week 6 was 0.60, which met the 0.40 threshold for anchor suitability. To determine the MWPC threshold for the MADRS, the lowest improvement category for the CGI-S with a large SES and a significant *P* value was identified. In this analysis, the 1-point improvement category on the CGI-S attained a significant within-group improvement with a large SES of 1.51 (*P*<.001). The 95% CIs for the “no change” and the 1-point improvement categories did not overlap. The median change in MADRS for the 1-point CGI-S improvement category was −8.0 (IQR −13.0 to −5.0) points, and this was outside of the 95% CI of the “no change” category. Using the CGI-S as an anchor, an 8-point improvement on the MADRS was recommended as the MWPC threshold.

**Table 2.** Within-groups uncollapsed anchor-based meaningful change derivation for the Montgomery-Åsberg Depression Rating Scale (MADRS) at week 6, using the Clinical Global Impression-Severity Scale (CGI-S) as an anchor (modified intent-to-treat, n=354).

Change level	Change correlation^a^	Correlation at baseline^b^	Values, n	Mean change (SD)	Median change (IQR)	95% CI	*P* value^c^	SES^d^ of change^e^
4-Point improvement	0.60	0.39	5	−21.4 (6.11)	−23.0 (−24.0 to −22.0)	−28.98 to −13.82	.0014^f^	3.57
3-Point improvement	N/A^g^	N/A	19	−18.4 (7.78)	−20.0 (−25.0 to −15.0)	−22.17 to −14.67	<.001	3.07
2-Point improvement	N/A	N/A	57	−14.0 (8.23)	−14.0 (−19.0 to −9.0)	−16.22 to −11.85	<.001	2.34
1-Point improvement	N/A	N/A	115	−9.1 (6.62)	−8.0 (−13.0 to −5.0)	−10.29 to −7.85	<.001	1.51
No change	N/A	N/A	109	−3.0 (6.76)	−2.0 (−7.0 to 2.0)	−4.30 to −1.73	<.001	0.50
1-Point worsening	N/A	N/A	7	4.0 (4.40)	3.0 (0 to 9.0)	−0.07 to 8.07	.053	0.67

a The correlation is the Spearman correlation coefficient between the change from baseline in continuous scores on the end point measure and the change from baseline in continuous scores on the anchor measure as presented in the table.

b The correlation is the Spearman correlation coefficient between baseline continuous scores on the MADRS and baseline continuous scores on the CGI-S.

c The *P* value for each individual change group is derived from a paired (within-samples) *t *test assessing the difference over time.

d SES: standardized effect size.

e SESs are calculated as the mean change divided by the SD at baseline. They are judged as: small=0.20, moderate=0.50, and large=0.80.

f *P*<.01.

g N/A: not applicable.

Using the PHQ-9 as an anchor, mean changes in MADRS scores from baseline to week 6 for each category monotonically decreased from worsening to improvement groups (Table 3). The correlation between change on the MADRS and change on the PHQ-9 (change correlation) at week 6 was 0.44, which met the 0.40 threshold for anchor suitability. The “improvement” category on the PHQ-9 attained a significant within-group improvement with an SES of 1.90 (*P*<.001). Comparison of the 95% CIs for the CGI-S “no change” category and the PHQ-9 “improvement” category showed no overlap. The median change for the PHQ-9 “improvement” category was −10 (IQR −17.0 to −5.0) points, which was outside of the 95% CI of the “no change” category. Using the PHQ-9 as an anchor, a 10-point improvement on the MADRS was recommended as the MWPC threshold.

**Table 3.** Within-groups collapsed anchor-based meaningful change derivation for the Montgomery-Åsberg Depression Rating Scale (MADRS) at week 6, using the Patient Health Questionnaire 9-Item Scale (PHQ-9) as an anchor (modified intent-to-treat, n=354).

Change level^a^	Change correlation^b^	Correlation at baseline^c^	Values, n	Mean change (SD)	Median change (IQR)	95% CI	*P* value^d^	SES^e^ of change^f^
Improvement	0.44	0.38	165	−11.4 (8.68)	−10.0 (−17.0 to −5.0)	−12.72 to −10.05	<.001	1.90
No change	N/A^g^	N/A	124	−5.0 (7.64)	−5.0 (−9.0 to 0)	−6.39 to −3.67	<.001	0.84
Worsening	N/A	N/A	9	−2.4 (7.60)	−1.0 (−6.0 to 2.0)	−8.29 to 3.40	.36	0.41

a Improvement=≥6-point improvement; worsening=≥6-point worsening.

b The correlation is the Spearman correlation coefficient between the change from baseline in continuous scores on the MADRS and the change from baseline in continuous scores on the PHQ-9 as presented in the table.

c The correlation is the Spearman correlation coefficient between baseline continuous scores on the end point measure and baseline continuous scores on the PHQ-9.

d The *P* value for each individual change group is derived from a paired (within-samples) *t *test assessing the difference over time.

e SES: standardized effect size.

f SESs are calculated as the mean change divided by the SD at baseline. They are judged as: small=0.20, moderate=0.50, and large=0.80.

g N/A: not applicable.

To support the MWPC threshold findings, the MADRS score change from baseline to week 6 was evaluated using CGI-S categories (Multimedia Appendix 1). The most frequently observed shifts were among participants who experienced a 1-point improvement from the CGI-S category of “moderate” at baseline to “mild” at week 6 and among participants who remained at “moderate” from baseline to week 6. For the 1-point improvement among participants who experienced a “moderate” to “mild” shift from baseline to week 6 on the CGI-S, the median change of the MADRS was −9.00 points. This median change is similar to the MADRS MWPC threshold findings of 8 points (CGI-S anchor) and 10 points (PHQ-9 anchor) derived in this analysis.

**Figure 1.**
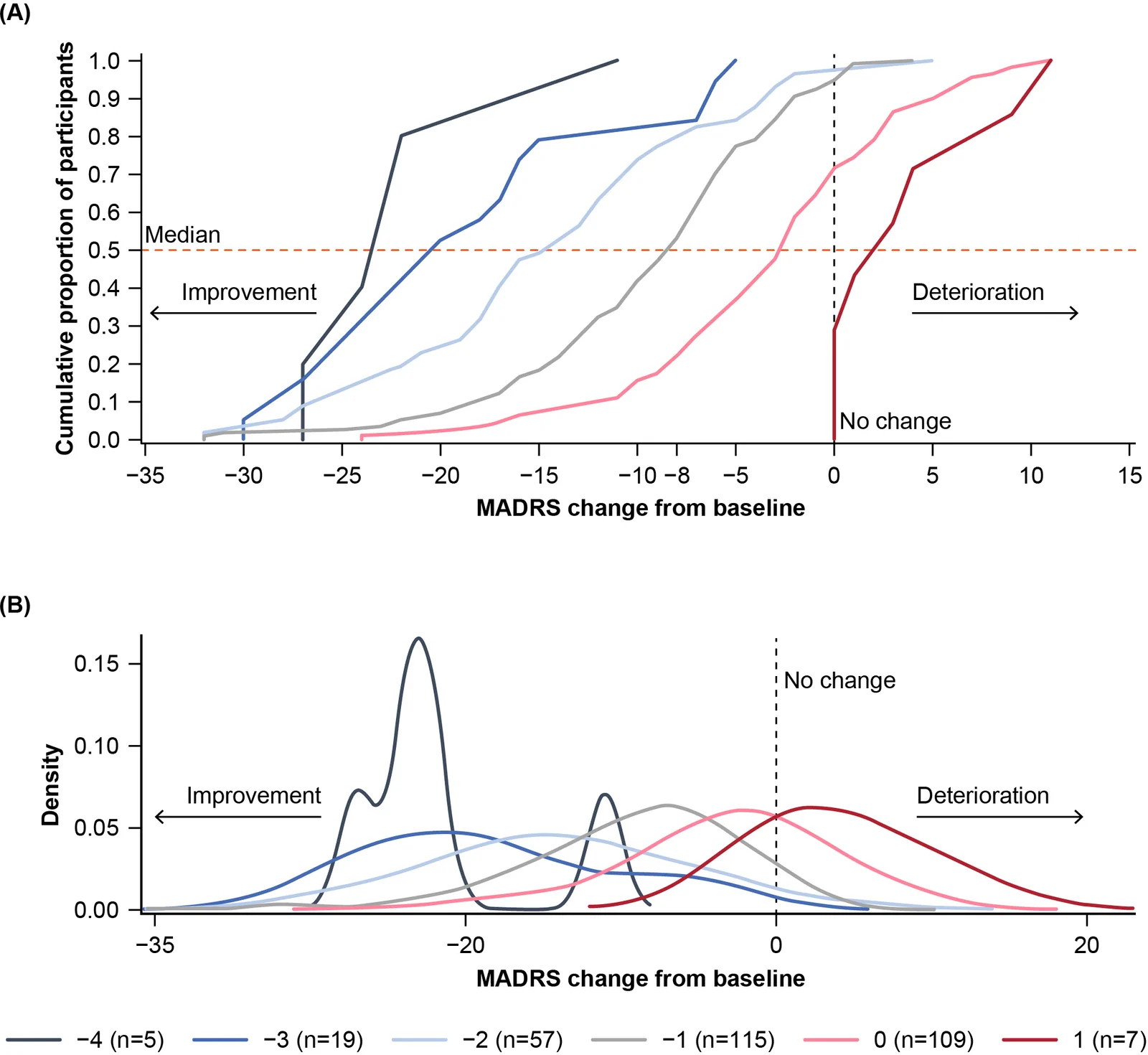
(A) eCDF and (B) PDF of the MADRS total score change from baseline to week 6 by uncollapsed change in CGI-S category (n=354). CGI-S: Clinical Global Impression-Severity Scale; eCDF: empirical cumulative distribution function; MADRS: Montgomery-Åsberg Depression Rating Scale; PDF: probability density function.

CDF and PDF curves were examined for the MADRS change from baseline to week 6. For the MADRS change score using CGI-S as an anchor, there was clear separation between the curves for all improvement, no change, and worsened CGI-S categories (Figure 1A). The median cut point for the 1-point improvement category on the CGI-S was approximately positioned at 8 points of MADRS improvement, which aligns with the MWPC threshold recommendation of −8 points.

When stratified by categories of change on the CGI-S, there was substantially greater improvement on the MADRS score observed at a 1-point change (improvement) on the CGI-S (Figure 1B). Categories of no change or worsening on the CGI-S were centered near 0 or at values of worsening on the MADRS, while all improvement categories of the CGI-S were centered at values of improvement.

**Figure 2.**
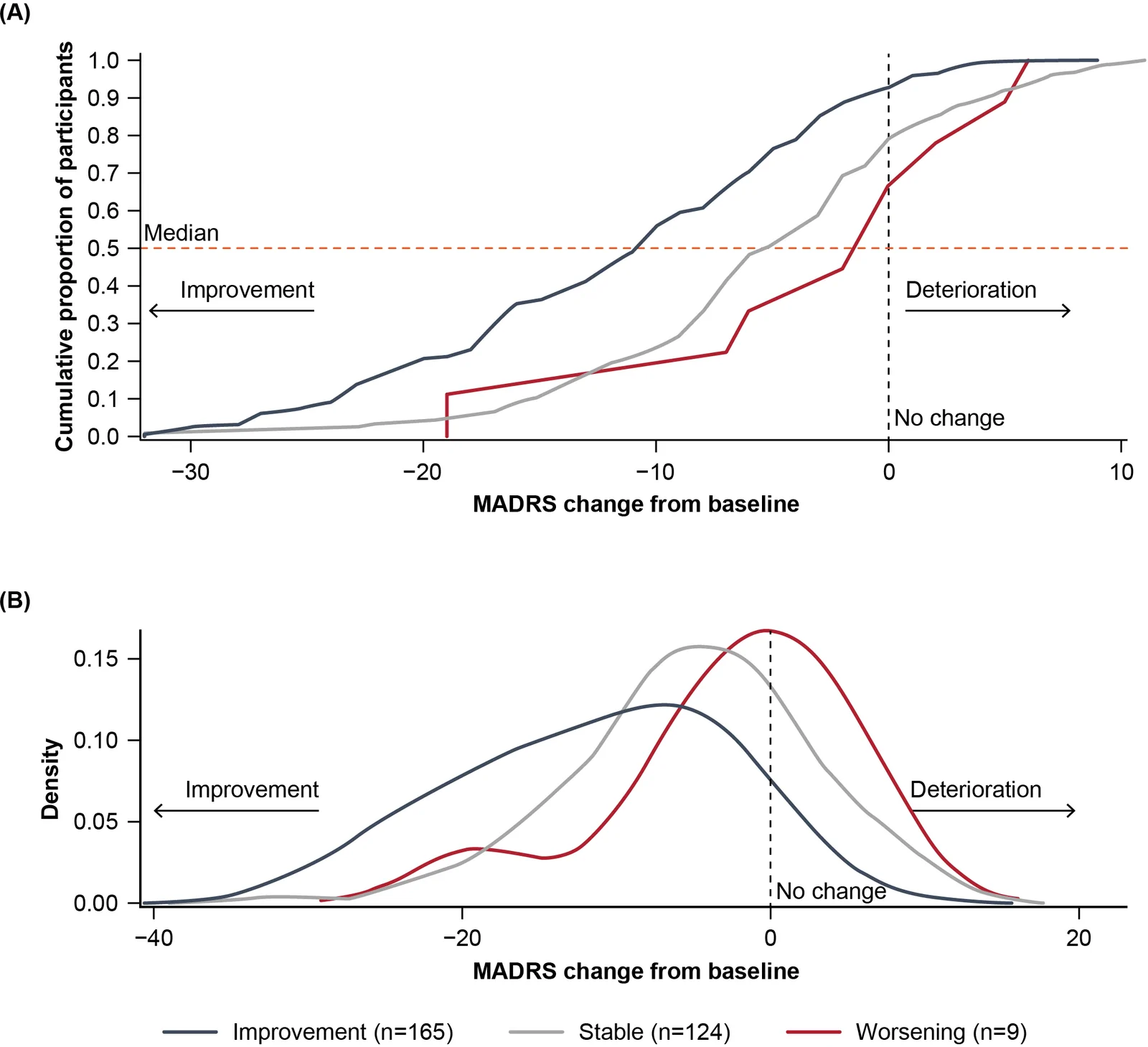
(A) eCDF and (B) PDF of the MADRS total score change from baseline to week 6 by collapsed change in PHQ-9 category (n=354). eCDF: empirical cumulative distribution function; MADRS: Montgomery-Åsberg Depression Rating Scale; PDF: probability density function; PHQ-9: Patient Health Questionnaire 9-Item Scale.

Similarly, for the MADRS change score using PHQ-9 as an anchor, there was clear separation between the curves for improvement, no change, and worsening PHQ-9 categories (Figure 2A). The median cut point for the improvement category on the PHQ-9 was approximately positioned at −11 points of improvement on the MADRS, which closely aligned with the MWPC threshold recommendation of −10 points. In the corresponding PDF curve (Figure 2B), there was slight overlap between the peak of the improvement category and the peak of the stable category. The worsening category on the PHQ-9 was centered at 0 change in MADRS score, while the stable and improvement categories of the PHQ-9 were centered at values of improvement on the MADRS.

To estimate group-based minimal important differences for the MADRS, the CGI-S and PHQ-9 were used as anchors, and adjacent improvement categories were compared (Table 4). The CGI-S 1-point improvement versus “no change” had an SES of 0.90 (*P*<.001). The mean difference of this comparison was −6.1 (SE 0.94) points, which represented the smallest change on the MADRS that can discriminate from no change based on the CGI-S. The PHQ-9 “improvement” versus “no change” categories had an SES of 0.77 (*P*<.001). The mean difference of this comparison was −6.4 (SE 0.98) points, which represented the smallest change on the MADRS that can discriminate from no change based on the PHQ-9. There was some overlap in the 95% CIs for the PHQ-9 category comparison of “no change” versus “worsening,” likely due to the small sample size in the worsening group (n=9).

**Table 4.** Between-groups anchor-based meaningful within-patient change derivation for the Montgomery-Åsberg Depression Rating Scale change from baseline to week 6 using the Clinical Global Impression-Severity Scale (CGI-S; uncollapsed categories) and Patient Health Questionnaire-9 Item Scale (PHQ-9; collapsed categories) as anchors (modified intent-to-treat, n=354).

Change in anchor response	Values, n	Mean difference (SE)^a^	95% CI	*P* value^b^	SES^c^ of change^d^
CGI-S					
4- versus 3-point improvement	5	−3.0 (3.53)	−9.93 to 3.97	.40	0.40
3- versus 2-point improvement	19	−4.4 (1.86)	−8.05 to −0.73	.02^e^	0.54
2- versus 1-point improvement	57	−5.0 (1.14)	−7.20 to −2.73	<.001	0.69
1-point versus no change	115	−6.1 (0.94)	−7.90 to −4.20	<.001	0.90
No change versus 1-point worsening	109	−7.0 (2.74)	−12.41 to −1.63	.011^e^	1.05
PHQ-9^f^					
Improvement versus no change	165	−6.4 (0.98)	−8.28 to −4.43	<.001	0.77
No change versus worsening	124	−2.6 (2.84)	−8.18 to 3.00	.36	0.34

a SE is presented for between-group comparisons.

b The *P* value for each individual comparison is derived from a between-group *t* test.

c SES: standardized effect size.

d SESs are calculated as the mean difference in change scores divided by the pooled SD of change. They are judged as: small=0.20, moderate=0.50, and large=0.80.

e *P*<.05.

f Improvement=≥6-point improvement; worsening=≥6-point worsening.

### Responder Analyses

**Figure 3.**
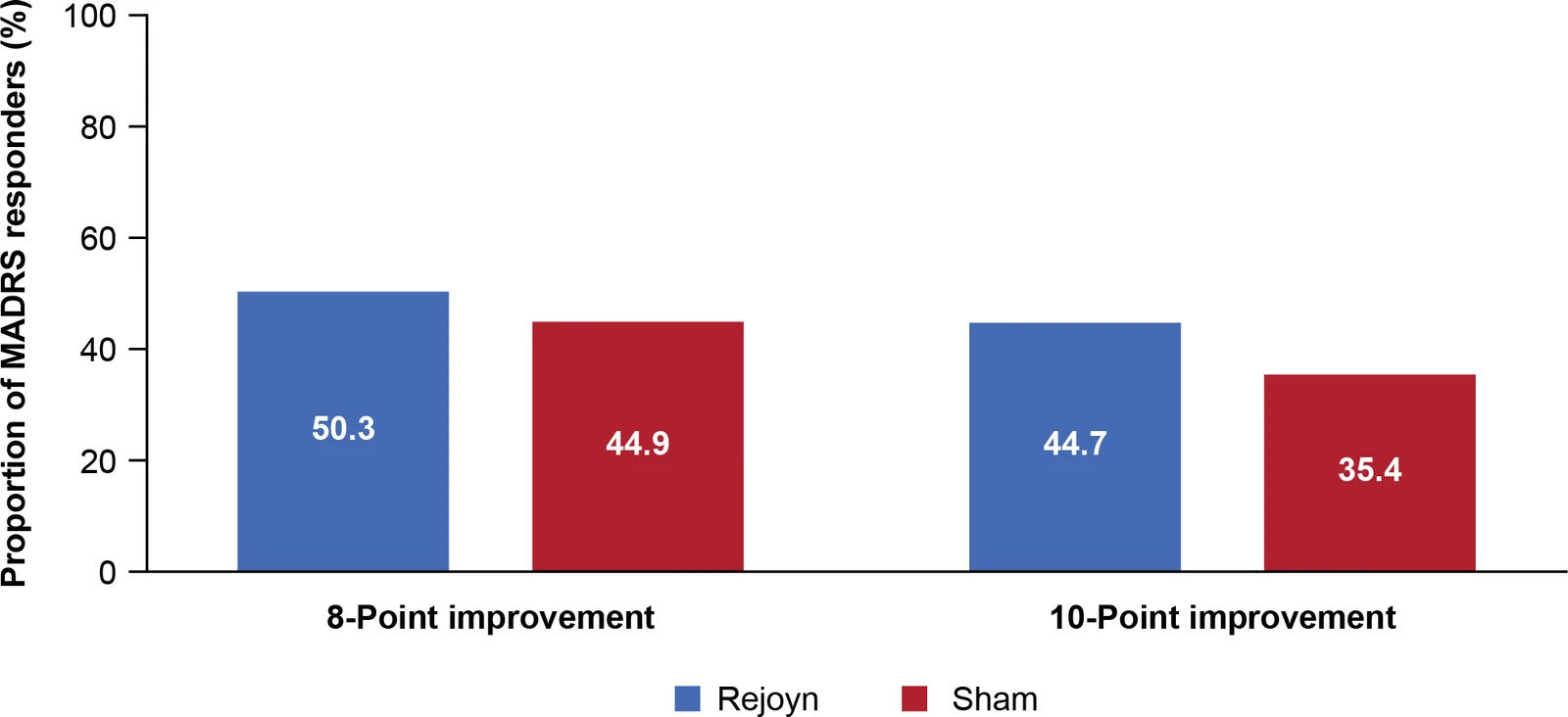
Proportions of MADRS responders at 8-Point and 10-Point MWPC improvement^a^ thresholds by treatment group at week 6 in the mITT (n=354) sample. ^a^Improvement was defined as a change in score from baseline that meets or exceeds the defined meaningful change threshold in the direction of improvement. All other patients were classified as not improved. MADRS: Montgomery-Åsberg Depression Rating Scale; mITT: modified intent-to-treat; MWPC: meaningful within-patient change.

**Figure 4.**
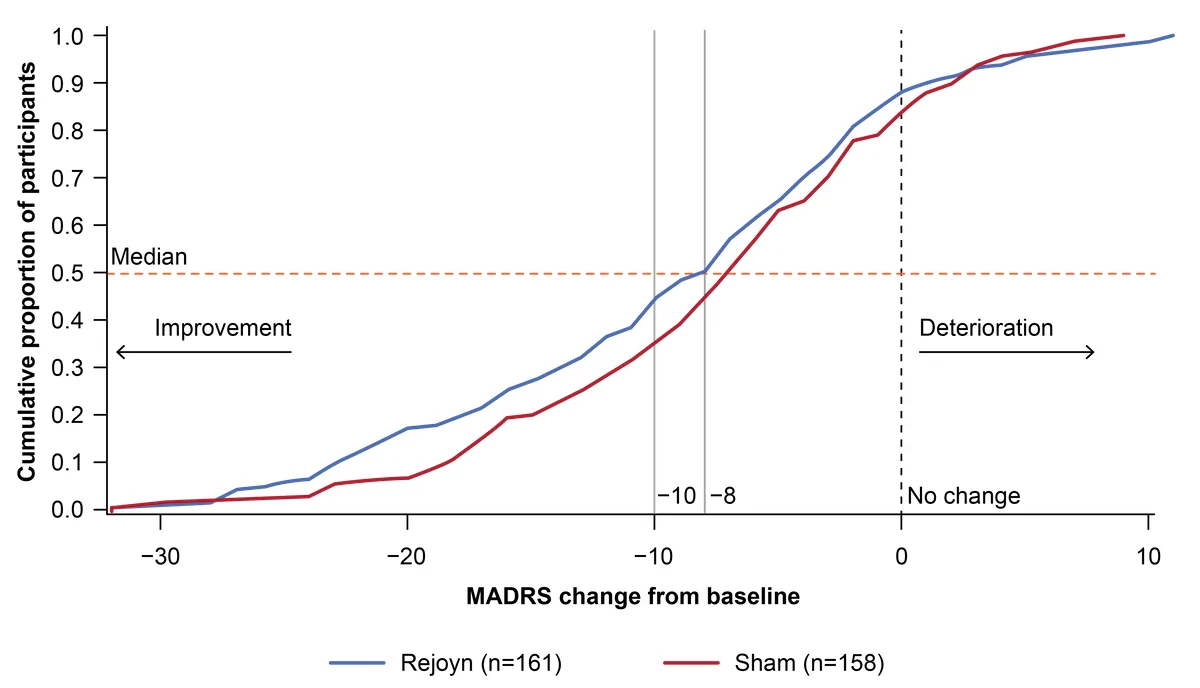
eCDF of the MADRS total score change from baseline to week 6 by treatment group (n=354). eCDF: empirical cumulative distribution function; MADRS: Montgomery-Åsberg Depression Rating Scale.

With both MWPC thresholds of 8-point and 10-point MADRS improvement, the distribution of response in the mITT sample favored the Rejoyn group over the sham group (Figure 3). Using the MWPC threshold of 8-point improvement for the MADRS (CGI-S anchor), 50.3% (81/161) and 44.9% (71/158) of participants in the Rejoyn and sham groups, respectively, improved from baseline to week 6. Compared with the sham group, participants in the Rejoyn group had 24% higher odds of meaningful improvement on the MADRS and were 12% more likely to experience meaningful improvement on the MADRS. Using the MWPC threshold of 10-point improvement for the MADRS (PHQ-9 anchor), 44.7% (72/161) and 35.4% (56/158) of patients in the Rejoyn and sham groups, respectively, improved from baseline to week 6. Compared with the sham group, participants in the Rejoyn group had 47% higher odds of meaningful improvement on the MADRS and were 26% more likely to experience meaningful improvement on the MADRS. Unblinded CDF curves of the change from baseline to week 6 on the MADRS showed separation between the 2 treatment groups, with the Rejoyn group showing a greater cumulative proportion of patients with at least 8-point or 10-point MADRS improvement compared with the sham group (Figure 4).

Results of the sensitivity analysis using the ITT sample were consistent with the results of the mITT responder analyses; nominal significance was reached for the 10-point MWPC threshold.

### Exploratory Analysis of MADRS Difference Between Treatment Arms

Using the raw, unadjusted MADRS scores, the mean MADRS score change from baseline to week 6 was −9.07 (SD 9.19) for the Rejoyn group and −7.53 (SD 8.19) for the sham group. The difference between the Rejoyn and sham groups in mean MADRS change from baseline to week 6 (−1.55) closely aligned with the treatment difference using the model-adjusted scores from the Mirai primary analysis (−1.78), which confirmed that there was a treatment difference between the Rejoyn and sham groups [9]. The mean MADRS improvement in the Rejoyn group surpassed the calculated MWPC of 8, whereas the mean improvement in the sham group did not. This indicated that more participants in the Rejoyn group experienced meaningful improvement compared with the sham group.

### Efficacy End Points in the ITT Sample

**Figure 5.**
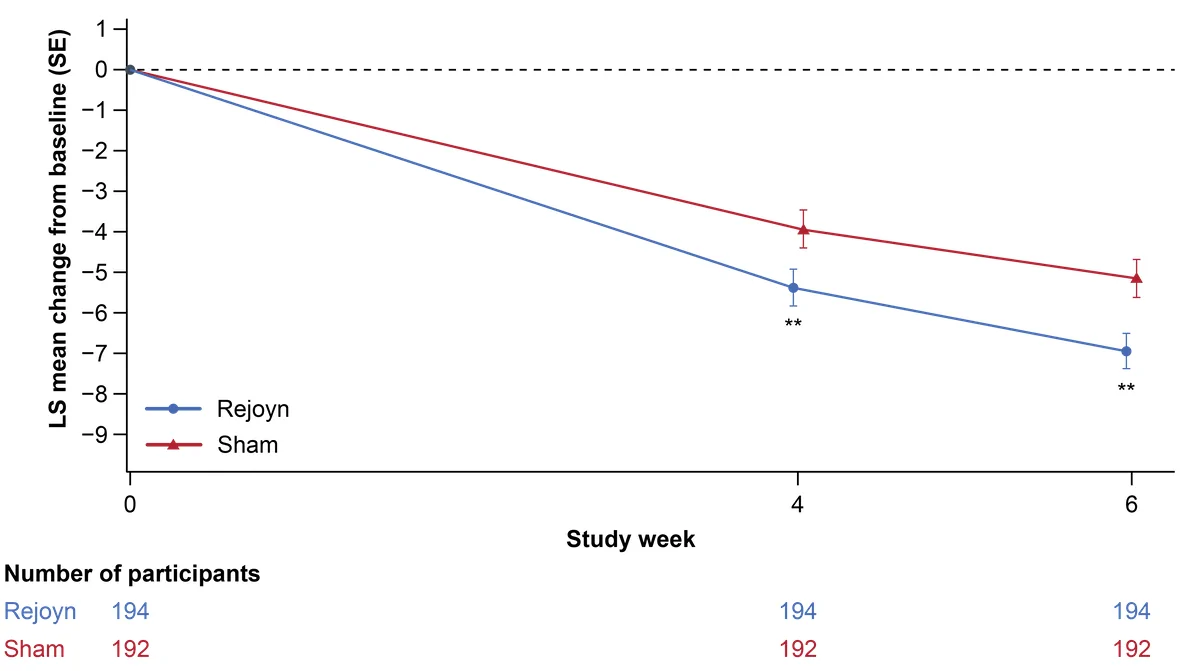
Mean change from baseline during the treatment period in PHQ-9 total score in the ITT sample. Error bars are LS mean±1 SE. ITT: intent-to-treat; LS: least squares; PHQ-9: Patient Health Questionnaire 9-Item Scale. **P<.01.

The primary efficacy outcome in Mirai was change in MADRS score from baseline to week 6 in the mITT sample; this, along with a prespecified supportive analysis using the ITT sample, and secondary outcomes in the mITT sample were all previously reported [9]. These data are reported along with other previously published values in Table 5 to compare with the post hoc analyses in the ITT sample [9]. Briefly, the least squares mean change from baseline to week 6 in MADRS total score in the ITT population was −8.78 (SE 0.79) in the Rejoyn group compared with −6.66 (SE 0.81) in the sham group (between-group difference −2.12; *P*=.02; 95% CI −3.93 to −0.32) [9]. Compared with the sham group, the Rejoyn group had greater full or partial response rate (*P*=.02), full response rate (*P*=.03), partial response rate (*P*=.56), and remission rate (*P*=.19; Table 5) [8]. The least squares mean change from baseline to week 6 in the PHQ-9 total score in the ITT population was −6.93 (SE 0.45) in the Rejoyn group compared with −5.15 (SE 0.47) in the sham group (between-group difference −1.78; *P*=.0012; 95% CI −2.85 to −0.71; Table 5 and Figure 5) [8]. The mean within-group change in the Rejoyn group represents a clinically meaningful and categorical improvement from “moderately severe” to “mild” [8,15,22]. In the sham group, the mean within-group change also represents a clinically meaningful change, associated with a categorical improvement from “moderately severe” to “moderate” [8,15].

**Table 5.** Mean change in efficacy end points from baseline to week 6 in the ITT^a^ and mITT^b^ samples.

	ITT sample				mITT sample			
	Rejoyn (n=194)	Sham (n=192)	Between-group difference	*P* value	Rejoyn (n=177)	Sham (n=177)	Between-group difference	*P* value
Outcome measure at week 6, least squares mean (SE)								
MADRS^c^	−8.78 (0.79)	−6.66 (0.81)	−2.12 (0.92)	.02^d^	−9.03 (0.73)	−7.25 (0.73)	−1.78 (0.93)	.06
PHQ-9^e^	−6.93 (0.45)	−5.15 (0.47)	−1.78 (0.55)	.0012^f^	−6.68 (0.45)	−5.10 (0.46)	−1.58 (0.53)	.003^f^
CGI-S^g^	−1.03 (0.09)	−0.74 (0.09)	−0.29 (0.10)	.004^f^	−1.06 (0.08)	−0.80 (0.08)	−0.26 (0.10)	.0098^f^
MADRS response rates at week 6 (%)								
Full or partial^h^	51.3	38.7	1.32^i^	.02^d^	48.3	37.5	1.27^i^	.0485^d^
Full^j^	30.4	20.2	1.49^i^	.03^d^	28.4	20.4	1.38^i^	.09
Partial^k^	20.9	18.6	1.14^i^	.56	19.9	17.0	1.15^i^	.53
Remission^l^	18.2	13.0	1.39^i^	.19	17.0	13.6	1.24^i^	.39

a ITT: intent-to-treat.

b mITT: modified intent-to-treat

c MADRS: Montgomery-Åsberg Depression Rating Scale.

d *P*<.05.

e PHQ-9: Patient Health Questionnaire 9-Item Scale.

f *P*<.01.

g CGI-S: Clinical Global Impressions-Severity Scale.

h ≥30% reduction from baseline at week 6.

i Relative risk defined by the ratio of response rate in Rejoyn group over the response rate in sham group.

j ≥50% reduction from baseline at week 6.

k ≥30%-50% reduction from baseline at week 6.

l ≥50% reduction from baseline and MADRS ≤10 at week 6.

**Figure 6.**
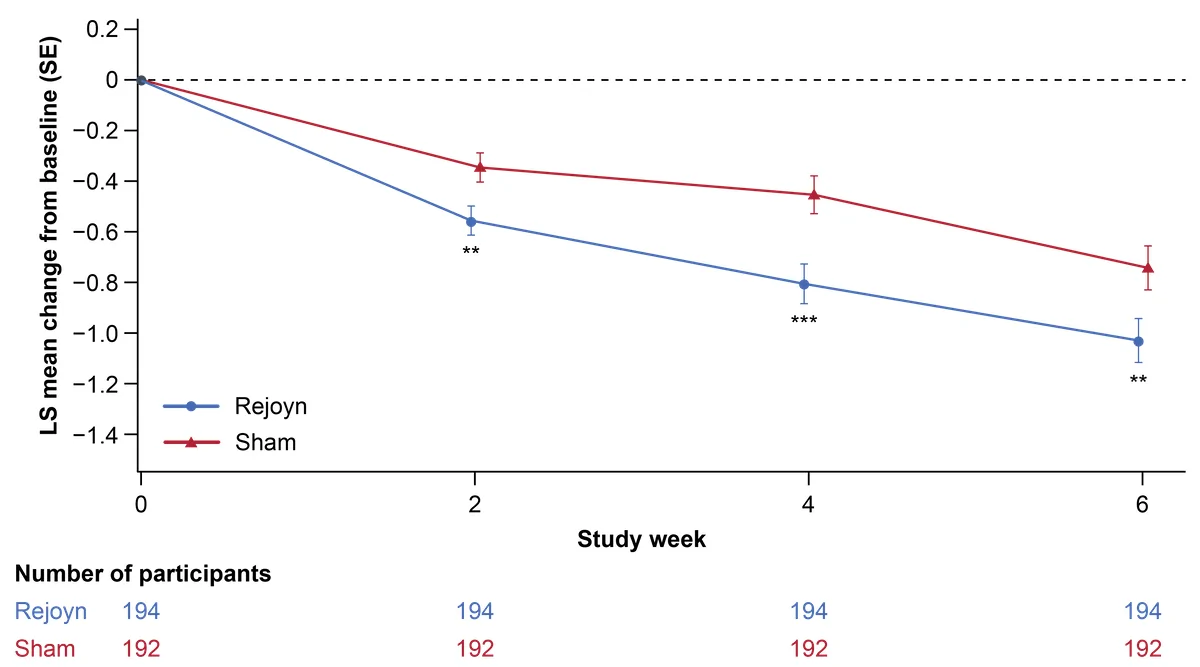
Mean change from baseline during the treatment period in CGI-S total score in the ITT sample. Error bars are LS mean±1 SE. CGI-S: Clinical Global Impression-Severity Scale; ITT: intent-to-treat; LS: least squares. **P<.01; ***P<.001.

The least squares mean change from baseline to week 6 for the CGI-S total score was −1.03 (SE 0.09) in the Rejoyn group compared with −0.74 (SE 0.09) in the sham group (between-group difference −0.29; *P*=.004; 95% CI −0.48 to −0.09; Table 5 and Figure 6) [8]. The mean within-group change in the Rejoyn group represents a clinically meaningful and categorical improvement from “moderately ill” to “mildly ill” [8,22]. The mean within-group change in the sham group is not clinically meaningful [8].

## Discussion

### Principal Findings

The totality of the findings of the post hoc analyses presented here are consistent with the prespecified analyses of Mirai (primary mITT and supportive ITT primary end point analyses), which found that Rejoyn was effective as an adjunctive treatment for MDD compared with sham [9]. The MWPC analyses offered a measure of meaningful change on the MADRS that may be more relevant to interpretation in clinical practice. This is also consistent with current regulatory standards regarding clinical meaningfulness of treatment effects for patients.

Using both clinician (CGI-S) and patient (PHQ-9) scales as part of an anchor-based approach, MWPC thresholds of −8 and −10 points on the MADRS were proposed. Applying these thresholds to the distribution of MADRS responders showed that change from baseline in depressive symptoms favored the Rejoyn group over the sham group. The expanded post hoc analyses of the ITT population, including sensitivity analyses using the derived MWPC thresholds, also favored the Rejoyn group over the sham group for response rates and PHQ-9 and CGI-S score change from baseline. The between-group analysis across the adjacent change categories indicated that a 6-point change was the smallest improvement on the MADRS that could discriminate from patients in the “no change” category from the “improvement” categories for the CGI-S. Taken together, the MWPC analyses allowed us to better contextualize and interpret the Mirai findings in routine daily practice, using various anchors from scales with known treatment differences to generate a range of thresholds for meaningful clinical change on the MADRS [8,15,16]. Providing such context using current methodologies is additionally important, because most apps for MDD that have been tested for effectiveness or safety in clinical trials did not use a robust control [9,23-26].

While there are a few recent studies calculating MWPC in the MADRS in MDD and treatment-resistant depression (TRD), there are none using a DTx. Hudgens et al [15] found an MWPC threshold of −10 for the MADRS using CGI-S as an anchor in a clinical trial (oral esketamine) population of participants with TRD, then applied that threshold to a second, similar population to compare meaningful differences in treatment groups. They also highlighted the effectiveness of the PHQ-9 in capturing overall symptom changes in patients with TRD and reported an MWPC of −6 [15]. In a pooled analysis of both populations, Turkoz et al [22] calculated clinically meaningful (corresponding to a 1-point change in the CGI-S) and clinically substantial (corresponding to a 2-point change in the CGI-S) changes for the MADRS and PHQ-9. They concluded that −6 points demonstrated “clinically meaningful,” and −12 points “clinically substantial” within-patient change over a given treatment period on the MADRS [22]. While not using currently preferred methods, Leucht et al [27] concluded from a review of 22 studies (mirtazapine for MDD) that an 8- to 9-point within-patient change corresponded to a 1-point change on the CGI-S. Although calculated from different populations using different treatment modalities, the similarities of findings in the literature on the MADRS MWPC and the utility of the PHQ-9 for depression support the findings of this post hoc study on the effectiveness of Rejoyn in Mirai.

### Limitations

Generally, the limitations to the original study and dataset are also limitations in these post hoc analyses [9]. These limitations include excluding older adult participants and having a majority female population; not assessing longer-term outcomes, although MDD is a chronic condition; a potentially reduced effect size with the use of a sham app as a control rather than a waitlist, which could result in an expectation of benefit; and while the EFMT algorithm was adaptive, CBT-based lessons and study visits were not adaptive or personalized to individual participants [9]. Except for the supportive analysis of the primary efficacy end point, these analyses were not prespecified or powered for statistical significance, and thresholds were derived post hoc; results should be interpreted with caution. An additional potential limitation is that oftentimes, MWPC analyses are calculated using multiple datasets, with the thresholds being derived from one set of data and then applied to a second set of data from a similar patient population. In this post hoc analysis, only a single set of data was used to derive the thresholds, which were then applied back to the same set of data; however, each scale was completed by different raters. MWPC thresholds are specific to the patient population studied; thus, generalizability of these results to a broader population would not be appropriate.

### Conclusions

The findings from the post hoc analyses presented here are consistent with the primary findings of the Mirai trial [9]. Post hoc analyses using current, preferred methods to calculate meaningful change thresholds allow for the findings of the Mirai study to be additionally viewed in the framework of clinical meaningfulness. Using the calculated thresholds of −8 (CGI-S) and −10 (PHQ-9) on the MADRS, Rejoyn was favored over sham for meaningful change from baseline in depressive symptoms. Findings from these analyses are supported by MADRS MWPC threshold analyses in the published literature [15,22,27]. When looking across the primary outcomes and post hoc data reported here, the totality of evidence converges to support Rejoyn as effective as an adjunctive treatment for MDD compared with sham [9].

## Supplementary material

10.2196/85037 Multimedia Appendix 1 Change on the Montgomery-Åsberg Depression Rating Scale by baseline and week 6 Clinical Global Impression-Severity Scale score (modified intent-to-treat, n=354).

10.2196/85037 Checklist 1 CONSORT checklist.

## Abbreviations

CBT: cognitive behavioral therapy
CDF: cumulative distribution function
CGI-S: Clinical Global Impression-Severity Scale
CONSORT: Consolidated Standards of Reporting Trials
DSM: Diagnostic and Statistical Manual of Mental Disorders
DTx: digital therapeutic
eCDF: empirical cumulative distribution function
EFMT: Emotional Faces Memory Task
FDA: US Food and Drug Administration
GAD-7: Generalized Anxiety Disorder 7-Item Scale
IRB: institutional review board
ITT: intent-to-treat
MADRS: Montgomery-Åsberg Depression Rating Scale
MDD: major depressive disorder
mITT: modified intent-to-treat
MWPC: meaningful within-patient change
PDF: probability density function
PHQ-9: Patient Health Questionnaire 9-Item Scale
SES: standardized effect size
TRD: treatment-resistant depression
