# Supplementary material for: Supporting the Primary Outcomes of the Mirai Trial for the Adjunctive Digital Therapeutic Rejoyn (CT-152) in the Treatment of Major Depressive Disorder: Meaningful Change Analysis in the Montgomery-Åsberg Depression Rating Scale
Source: JMIR Form Res. 2026 Sep 21;10:e85037. doi: 10.2196/85037 (PMC13592388; doi:10.2196/85037)
Supplement: Supplementary file 1 [file formative-v10-e85037-s001.docx]

**Multimedia Appendix 1**. Change on the MADRS by baseline and week 6 CGI-S score (mITT, n=354)

|  | **CGI-S response at week 6** | | | | | |
| --- | --- | --- | --- | --- | --- | --- |
| **CGI-S response at baseline** | | **Normal  (1 point)** | **Borderline  (2 points)** | **Mild  (3 points)** | **Moderate  (4 points)** | **Marked  (5 points)** |
| Mild  (3 points) | n | 1 | 2 |  |  |  |
|  | Mean (SD) | −23.00 (NA) | −6.50 (4.95) |  |  |  |
|  | Median | −23.00 | −6.50 |  |  |  |
|  | 95% CI |  | −50.97, 37.97 |  |  |  |
| Moderate  (4 points) | n | 9 | 27 | 79 | 91 | 7 |
|  | Mean (SD) | −23.22 (5.04) | −14.82 (8.12) | −10.27 (6.98) | −3.33 (6.78) | 4.00 (4.40) |
|  | Median | −23.00 | −15.00 | −9.00 | −3.00 | 3.00 |
|  | 95% CI | −27.10, −19.35 | −18.03, −11.60 | −11.83, −8.70 | −4.74, −1.92 | −0.07, 8.07 |
| Marked  (5 points) | n | 3 | 9 | 25 | 34 | 18 |
|  | Mean (SD) | −24.00 (2.65) | −15.00 (7.25) | −14.76 (7.40) | −6.44 (4.97) | −1.44 (6.62) |
|  | Median | −23.00 | −17.00 | −16.00 | −6.00 | 0.00 |
|  | 95% CI | −30.57, −17.43 | −20.57, −9.43 | −17.82, −11.71 | −8.18, −4.71 | −4.74, 1.85 |
| Severe  (6 points) | n |  | 2 | 1 | 4 |  |
|  | Mean (SD) |  | −17.50 (9.19) | −6.00 (NA) | −2.00 (5.10) |  |
|  | Median |  | −17.50 | −6.00 | −3.00 |  |
|  | 95% CI |  | −100.09, 65.09 |  | −10.11, 6.11 |  |

^a^This analysis consists of participants who have observed scores (ie, nonmissing responses) in both the CGI-S and MADRS at both baseline and week 6.

CI, confidence interval; CGI-S, Clinical Global Impression-Severity scale; MADRS, Montgomery-Åsberg Depression Rating Scale; mITT, modified intent-to-treat; NA, not applicable; SD, standard deviation.
