# Supplementary material for: Supporting the Primary Outcomes of the Mirai Trial for the Adjunctive Digital Therapeutic Rejoyn (CT-152) in the Treatment of Major Depressive Disorder: Meaningful Change Analysis in the Montgomery-Åsberg Depression Rating Scale
Source: JMIR Form Res. 2026 Sep 21;10:e85037. doi: 10.2196/85037 (PMC13592388; doi:10.2196/85037)
Supplement: Supplementary file 2 [file formative-v10-e85037-s002.docx]

**Checklist 1**. CONSORT 2025 checklist of information to include when reporting a randomized trial

| **Section / Topic** | **Item number** | **CONSORT 2025 checklist item description** | **Section reported** |
| --- | --- | --- | --- |
| **Title and abstract** | | |  |
| Title and structured abstract | 1a | Identification as a randomized trial | Title – analysis type identified |
|  | 1b | Structured summary of the trial design, methods, results, and conclusions | Abstract |
| **Open science** | | |  |
| Trial registration | 2 | Name of trial registry, identifying number (with URL) and date of registration | Abstract, Methods – Study Design |
| Protocol and statistical analysis plan | 3 | Where the trial protocol and statistical analysis plan can be accessed | Methods – Study Design |
| Data sharing | 4 | Where and how the individual de-identified participant data (including data dictionary), statistical code and any other materials can be accessed | Data Availability |
| Funding and conflicts of interest | 5a | Sources of funding and other support (eg, supply of drugs), and role of funders in the design, conduct, analysis and reporting of the trial | Acknowledgments, Funding Statement |
|  | 5b | Financial and other conflicts of interest of the manuscript authors | Author Contributions |
| **Introduction** | | |  |
| Background and rationale | 6 | Scientific background and rationale | Introduction |
| Objectives | 7 | Specific objectives related to benefits and harms | Introduction, Methods – Statistical Analysis |
| **Methods** | | |  |
| Patient and public involvement | 8 | Details of patient or public involvement in the design, conduct and reporting of the trial | NA |
| Trial design | 9 | Description of trial design including type of trial (eg, parallel group, crossover), allocation ratio, and framework (eg, superiority, equivalence, non-inferiority, exploratory) | Methods – Study Design |
| Changes to trial protocol | 10 | Important changes to the trial after it commenced including any outcomes or analyses that were not prespecified, with reason | NA – see protocol and primary publication [1, 2] |
| Trial setting | 11 | Settings (eg, community, hospital) and locations (eg, countries, sites) where the trial was conducted | Methods – Study Design, Methods – Participants and Recruitment |
| Eligibility criteria | 12a | Eligibility criteria for participants | Methods – Participants and Recruitment |
|  | 12b | If applicable, eligibility criteria for sites and for individuals delivering the interventions (eg, surgeons, physiotherapists) | NA |
| Intervention and comparator | 13 | Intervention and comparator with sufficient details to allow replication. If relevant, where additional materials describing the intervention and comparator (eg, intervention manual) can be accessed | Methods – Study Intervention |
| Outcomes | 14 | Pre-specified primary and secondary outcomes, including the specific measurement variable (eg, systolic blood pressure), analysis metric (eg, change from baseline, final value, time to event), method of aggregation (eg, median, proportion), and time point for each outcome | Methods – Statistical Analysis for post hoc analyses conducted |
| Harms | 15 | How harms were defined and assessed (eg, systematically, non-systematically) | NA |
| Sample size | 16a | How sample size was determined, including all assumptions supporting the sample size calculation | NA – see protocol and primary publication [1, 2] |
|  | 16b | Explanation of any interim analyses and stopping guidelines | NA – see protocol and primary publication [1, 2] |
| Randomization: |  |  |  |
| Sequence generation | 17a | Who generated the random allocation sequence and the method used | NA – see protocol and primary publication [1, 2] |
|  | 17b | Type of randomization and details of any restriction (eg, stratification, blocking and block size) | NA – see protocol and primary publication [1, 2] |
| Allocation concealment mechanism | 18 | Mechanism used to implement the random allocation sequence (e.g., central computer/telephone; sequentially numbered, opaque, sealed containers), describing any steps to conceal the sequence until interventions were assigned | NA – see protocol and primary publication [1, 2] |
| Implementation | 19 | Whether the personnel who enrolled and those who assigned participants to the interventions had access to the random allocation sequence | NA |
| Blinding | 20a | Who was blinded after assignment to interventions (eg, participants, care providers, outcome assessors, data analysts) | Methods – Statistical Analysis; also see protocol and primary publication [1, 2] |
|  | 20b | If blinded, how blinding was achieved and description of the similarity of interventions | Methods – Study Intervention; also see protocol and primary publication [1, 2] |
| Statistical methods | 21a | Statistical methods used to compare groups for primary and secondary outcomes, including harms | NA – see protocol and primary publication [1, 2] |
|  | 21b | Definition of who is included in each analysis (eg, all randomized participants), and in which group | Methods – Statistical Analysis; see protocol and primary publication [1, 2] |
|  | 21c | How missing data were handled in the analysis | NA – see protocol and primary publication [1, 2] |
|  | 21d | Methods for any additional analyses (eg, subgroup and sensitivity analyses), distinguishing prespecified from post-hoc | Methods – Statistical Analysis |
| **Results** | | |  |
| Participant flow, including flow diagram | 22a | For each group, the numbers of participants who were randomly assigned, received intended intervention, and were analyzed for the primary outcome | Table 1; also see protocol and primary publication [1, 2] |
|  | 22b | For each group, losses and exclusions after randomization, together with reasons | Table 1; also see protocol and primary publication [1, 2] |
| Recruitment | 23a | Dates defining the periods of recruitment and follow-up for outcomes of benefits and harms | NA – see protocol and primary publication [1, 2] |
|  | 23b | If relevant, why the trial ended or was stopped | NA |
| Intervention and comparator delivery | 24a | Intervention and comparator as they were actually administered (e.g., where appropriate, who delivered the intervention/comparator, how participants adhered, whether they were delivered as intended [fidelity]) | NA – see protocol and primary publication [1, 2] |
|  | 24b | Concomitant care received during the trial for each group | Methods – Participants and Recruitment |
| Baseline data | 25 | A table showing baseline demographic and clinical characteristics for each group | NA – see primary publication [2] |
| Numbers analyzed,  outcomes and estimation | 26 | For each primary and secondary outcome, by group:   - the number of participants included in the analysis - the number of participants with available data at the outcome time point - result for each group, and the estimated effect size and its precision (such as 95% confidence interval) - for binary outcomes, presentation of both absolute and relative effect size | NA – see primary publication [2] |
| Harms | 27 | All harms or unintended events in each group | NA – see primary publication [2] |
| Ancillary analyses | 28 | Any other analyses performed, including subgroup and sensitivity analyses, distinguishing pre-specified from post-hoc | Results, all sections |
| **Discussion** | | |  |
| Interpretation | 29 | Interpretation consistent with results, balancing benefits and harms, and considering other relevant evidence | Discussion – Principal Findings |
| Limitations | 30 | Trial limitations, addressing sources of potential bias, imprecision, generalizability, and, if relevant, multiplicity of analyses | Discussion – Limitations |

CONSORT, Consolidated Standards of Reporting Trials; NA, not applicable.

Table adapted from: Hopewell S, Chan AW, Collins GS, Hróbjartsson A, Moher D, Schulz KF, et al. CONSORT 2025 Statement: updated guideline for reporting randomised trials. BMJ. 2025; 388:e081123. <https://dx.doi.org/10.1136/bmj-2024-081123>.

© 2025 Hopewell et al. This is an Open Access article distributed under the terms of the Creative Commons Attribution License (<https://creativecommons.org/licenses/by/4.0/>).

**Table references**

1. Rothman B, Slomkowski M, Speier A, Rush AJ, Trivedi MH, Lawson E, et al. Evaluating the Efficacy of a Digital Therapeutic (CT-152) as an Adjunct to Antidepressant Treatment in Adults With Major Depressive Disorder: Protocol for the MIRAI Remote Study. JMIR Res Protoc. 2024 Aug 20;13:e56960. PMID: 39163592. doi: 10.2196/56960.
2. Rothman B, Slomkowski M, Speier A, Rush AJ, Trivedi MH, Lakhan SE, et al. A digital therapeutic (CT-152) as adjunct to antidepressant medication: A phase 3 randomized controlled trial (the Mirai study). J Affect Disord. 2025 Nov 1;388:119409. PMID: 40378969. doi: 10.1016/j.jad.2025.119409.
